# Toxic marine microalgae and shellfish poisoning in the British isles: history, review of epidemiology, and future implications

**DOI:** 10.1186/1476-069X-10-54

**Published:** 2011-06-06

**Authors:** Stephanie L Hinder, Graeme C Hays, Caroline J Brooks, Angharad P Davies, Martin Edwards, Anthony W Walne, Mike B Gravenor

**Affiliations:** 1 Institute of Life Science, Swansea University, Singleton Park, Swansea, SA2 8PP, UK; 2 Institute of Environmental Sustainability, Swansea University, Singleton Park, Swansea SA2 8PP, UK; 3 Health Information Research Unit (HIRU), Swansea University, Singleton Park, Swansea, SA2 8PP, UK; 4 SAHFOS, The Laboratory, Citadel Hill, Plymouth, PL1 2PB, UK; 5 Marine Institute, University of Plymouth, Drake Circus, Plymouth, PL4 8AA, UK

## Abstract

The relationship between toxic marine microalgae species and climate change has become a high profile and well discussed topic in recent years, with research focusing on the possible future impacts of changing hydrological conditions on Harmful Algal Bloom (HAB) species around the world. However, there is very little literature concerning the epidemiology of these species on marine organisms and human health. Here, we examine the current state of toxic microalgae species around the UK, in two ways: first we describe the key toxic syndromes and gather together the disparate reported data on their epidemiology from UK records and monitoring procedures. Secondly, using NHS hospital admissions and GP records from Wales, we attempt to quantify the incidence of shellfish poisoning from an independent source. We show that within the UK, outbreaks of shellfish poisoning are rare but occurring on a yearly basis in different regions and affecting a diverse range of molluscan shellfish and other marine organisms. We also show that the abundance of a species does not necessarily correlate to the rate of toxic events. Based on routine hospital records, the numbers of shellfish poisonings in the UK are very low, but the identification of the toxin involved, or even a confirmation of a poisoning event is extremely difficult to diagnose. An effective shellfish monitoring system, which shuts down aquaculture sites when toxins exceed regularity limits, has clearly prevented serious impact to human health, and remains the only viable means of monitoring the potential threat to human health. However, the closure of these sites has an adverse economic impact, and the monitoring system does not include all toxic plankton. The possible geographic spreading of toxic microalgae species is therefore a concern, as warmer waters in the Atlantic could suit several species with southern biogeographical affinities enabling them to occupy the coastal regions of the UK, but which are not yet monitored or considered to be detrimental.

## Introduction

Within the UK, several toxic marine microalgae species, where some species are also known as Harmful Algal Bloom (HAB) species are present throughout the coastal regions. These species produce biotoxins, which are ingested by filter feeding organisms, accumulating within their flesh [1]. These toxins gradually get transferred to the higher trophic levels within the food web, posing a threat to human health, if the shellfish is consumed [2-4].

In UK waters, an effective shellfish monitoring system acts as a sentinel, and has prevented serious impact on human health but this has led to long-term closures of fisheries, with severe economic consequences [5]. The shellfish industry is an important aspect of the economy in the UK; with shellfish contributing to 42% of UK landings, and with the shellfish industry worth £267.1 million in 2008 [6]. There have been several in-depth reviews concerning the toxins produced by these toxic microalgae species, and the future implication of climate change on these species composition and potential alterations in locations. However, despite many closures of fisheries there is very little literature concerning the epidemiology of adverse events on human health. Generally, information regarding the acute manifestations of these illnesses is greatly under reported, and species involved not even identified. With the changing hydrological conditions and the general idea that toxic microalgae species are increasing in their geographical location and frequency [7,8], it is important to determine the possible future threat in the UK.

Here, we address the problem in two ways: first we describe the key toxic syndromes and gather together the disparate data on their epidemiology from sporadic UK records and monitoring procedures. We describe the ecology of the main species, including their seasonal patterns, global distribution and how these might be affected by climate change. Second, using the Health Information Research Unit for Wales (HIRU), all NHS hospital admissions and GP records involving shellfish toxins were identified within Wales, and the implication for monitoring shellfish poisoning in the UK are discussed.

## Toxic Syndromes

There are around 60 to 80 toxic marine microalgae species throughout the world, with dinoflagellates accounting for 75% of all such species [9]. The examination of toxins from this large and diverse group of dinoflagellates has lead to the identification of five major seafood poisoning categories [1,9]: paralytic shellfish poisoning (PSP), neurotoxic shellfish poisoning (NSP), diarrhetic shellfish poisoning (DSP), ciguatera fish poisoning (CFP), and a newly identified azaspiracid poisoning (AZP). Diatom species have also been identified as producing toxins causing Amnesic Shellfish Poisoning (ASP).

Within UK waters, only PSP, DSP, AZP and ASP are present, with each toxin syndrome having a different lethal dose, onset and duration time, and a range of symptoms. A review of each toxin and symptoms involved can be found in Table 1. There have been very few cases of NSP and CFP reported in the UK, and those that have occurred arose from imported contaminated fish [10,11]. Therefore we have not examined these two toxins syndromes.

**Table 1:** Toxin syndromes and symptoms within UK waters

	Toxin	Causal species	Symptoms	References
PSP	Saxitoxin and gonyautoxin	*Alexandrium spp*. *Gymnodinium spp*. *Pyrodinium spp*.	Tingling and numbness Drowsiness Incoherence **In high doses **- respiratory arrest or cardiovascular shock or death	[1,11,26,52]

DSP	Okadaic acid and Dinophysis toxin (1,2 and3)	*Dinophysis spp*. *Prorcentrum spp*.	Nausea Vomiting Diarrhoea Abdominal cramps	[1,26,53,54]
			
	Pectenotoxin	*Dinophysis spp*.		
			
	Yessotoxin	*Gonyaulax spinifera* *Lingulodinium polyedrum* *Protoceratium reticulatum*	**In high doses **- dehydration and shock	

AZP	Azaspiracids	*Azadinium spinosum*	Nausea Vomiting Diarrhoea Abdominal cramps	[22]

ASP	Domonic acid	*Pseudo-nitzschia*	Nausea Vomiting Diarrhoea Abdominal cramps Loss of short term memory	[11,26,55]

### UK Monitoring Procedures

Legislative requirements are implemented to monitor shellfish to ensure human consumer protection and to control the risk of shellfish poisoning [12]. In 2001, the Food Standards Agency (FSA) took responsibility for becoming a National Reference Laboratory (NRL) for the monitoring of marine biotoxins within the UK by collecting and analyzing samples of shellfish and water from around the harvesting regions [13,14]. Water samples are tested twice monthly for the presence of toxic algae and shellfish are tested on a monthly basis for the presence of toxins [13]. The number of water samples, shellfish flesh sampled and the number of contributing harvesting areas in Wales and England from 1999-2009 are shown in Table 2. Toxin threshold values and water action limits have been produced (Table 3) and maximum acceptable limits set [15]. If toxins are shown to exceed these limits, the FSA communicate to the local authority of the relevant infected beds, who can impose a temporary prohibition order, which closes the beds to harvesting until daily tests have returned negative results for two consecutive weeks [15]. To ensure the safety of the shellfish placed on market, the Food Business Operators (FBOs) are required under regulation (EC) No 853/2004 to monitor the levels of biotoxins and ensure they do not exceed regulatory limits [16].

**Table 2:** The number of shellfish flesh samples, water samples and the number of active classified shellfish production and relaying areas collected by CEFAS from 1999-2009 in Wales and England

	Shellfish Testing		Water Samples		
		
	No. of Samples	No. of active classified shellfish production and relaying areas	No. of Samples	No. of active classified shellfish production and relaying areas	Reference
**April 1999 - March 2000**	1017	25	320	19	[49]

**April 2000 - March 2001**	703	34	350	20	[56]

**April 2001 - March 2002**	1326	67	195	23	[57]

**April 2002 - March 2003**	1529	64	1529	23	[58]

**April 2003 - March 2004**	1326	66	388	23	[59]

**April 2004 - March 2005**	949	64	314	21	[50]

**April 2005 - March 2006**	1143	64	737	61	[60]

**April 2006 - March 2007**	941	64	879	54	[61]

**April 2007 - March 2008**	1163	64	1122	54	[62]

**April 2008 - March 2009**	1059	66	1079	56	[51]

**Table 3:** Regulative limits of the maximum toxin levels of the four major seafood poisoning categorises that is allowed to be present in shellfish and the action limit of four genera in water samples within the UK

Regulative limits of maximum toxin level within the UK		
**Type of Shellfish Poisoning**	**Toxin**	**Maximum Level of Toxin**

PSP	STX in bivalve molluscs	80 mg STX eg/100 g of meat [63]

DSP	OA, DTXs and PTXs in edible tissues (whole of any part edible separately) of molluscs, echinoderms, tunicates and marine gastropods	160 mg OA equivalents/kg [64]
	
	YTX in edible tissues (whole of any part edible separately) of molluscs, echinoderms, tunicates and marine gastropods.	1 mg YTX equivalents/kg [64]

AZP	AZP toxins in bivalve molluscs, echinoderms, tunicates and marine gastropods (whole body or any part edible separately)	160 μg/kg [64]

ASP	DA toxin content in the edible parts of molluscs (the entire body or any part edible separately).	20 mg/kg [65]

		

**Water Sampling, maximum species abundance in the UK**		

	**Species**	**Action Limit (cells/litre)**

PSP	*Alexandrium spp*.	Present [62]

DSP	*Dinophysis spp*.	100 [62]
	
	*Prorocentrum spp*.	100 [62]

ASP	*Pseudo-nitzschia spp*.	150,000 [62]

**Figure 1:**
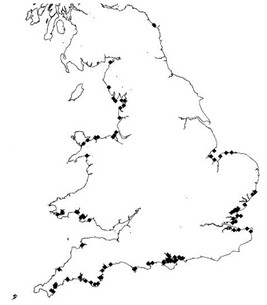
**English and Welsh flesh sampling locations - FSA Biotoxin monitoring programme 1^st ^April 2008 to 31st March 2009 (adapted with permission from CEFAS **[51]).

The statutory laboratory testing methods for shellfish toxins vary throughout the UK. The Centre for Environment, Fisheries and Aquaculture Science (CEFAS) monitors PSP and DSP in England, Wales and Scotland, and ASP in England and Wales [12]. In Northern Ireland, the Department for Agriculture and Rural Development (DARD) has conducted the testing program [15]. The method currently specified by European Food Safety legislation for Official Control testing for PSP and DSP are mouse bioassays (MBAs) based on the protocol of Yasumoto et al., [15,17], whereby shellfish extract is injected into mice, followed by observation of the survival time [15]. The bioassay results are compared to the threshold limits (Table 3) to determine if the toxin level is exceeded, which could result in the closure of the bed [12]. However, with the discovery of a range of novel lipophilic compounds associated with DSP toxins, Pectenotoxins (PTXs) and (Yessotoxins) YTXs, which give a positive result in the DSP MBAs, this approach is considered inadequate in scale for these toxins (EC) No 225/2002 [18]. This and the fact that the MBA method has considerable ethical objections, has lead to a demand to use alternative approaches [12,19]. Commission regulation (EC) No. 2074/2005 was revised to allow other detection approaches to be used, as alternative or supplementary methods to the MBAs, as long as they are as effective and EC 853/2004 valid [20]. Since 2006, a high performance liquid chromatography (HPLC) method has been used as a qualitative screen with the MBA used to provide a quantitative result from HPLC positive samples [14]. AZP toxins are harder to detect, as the toxins are not confined to the digestive glands but are distributed throughout all tissues, rendering the MBAs method inefficient [21,22]. Several EU Member states are currently using Liquid Chromatography-Mass Spectrometry (LC-MS) and Liquid Chromatography- UV detection (LC-UV) methods [20] to measure and determine toxin concentrations of AZP, ASP, DSP, and DSP lipophilic toxins (PTXs, YTXs) [22-24]. The success of the toxin detection and quantification of the LC-MS method is due to its efficient toxin separation, high sensitivity (lower limits of detection than MBA), high selectivity, and accurate and precise quantification [19]. However, LC-MS cannot detect mixtures of two different toxins, unlike the MBA method. Figure 1, show the location of samples collected in Wales and England during April 2008 to March 2009, and Tables 4 and 5 show the CEFAS shellfish testing and water sample results from April 1990- March 2009.

**Table 4:** CEFAS shellfish flesh results for the toxins, DSP, PSP and ASP from 1999-2009 in Wales and England

	DSP			PSP			ASP			
	
	No. samples tested	% Present	No. areas effected	No. samples tested	% Present	No. areas effected	No. samples tested	% Present	No. areas effected	Reference
**April 1999 - March 2000**	302	15 (5.0%)	5	370	5 (1.4%)	3	345	21 (6.1%)	21	[49]

**April 2000 - March 2001**	579	115 (19.9%)	35	438	8 (1.8%)	3	365	32 (8.8%)	32	[56]

**April 2001 - March 2002**	1173	166 (14.2%)	5	774	5 (0.6%)	4	768	10 (1.3%)	3	[57]

**April 2002 - March 2003**	1342	203 (15.1%)	20	981	12 (1.2%)	2	902	10 (1.1%)	7	[58]

**April 2003 - March 2004**	1128	148 (13.1%)	15	873	1 (0.1%)	1	818	11 (1.3%)	7	[59]

**April 2004 - March 2005**	847	14 (1.6%)	8	876	2 (0.2%)	2	841	8 (1.0%)	5	[50]

**April 2005 - March 2006**	1023	4 (0.4%)	3	1085	4 (0.4%)	2	1004	9 (0.9%)	2	[60]

	**LTs**			**PST**			**AST**			

**April 2006 - March 2007**	821	5 (0.6%)	3	892	8 (0.9%)	3	823	21 (2.6%)	7	[61]

**April 2007 - March 2008**	1024	9 (0.9%)	5	1099	3 (0.3%)	1	985	6 (0.6%)	5	[62]

**April 2008 - March 2009**	926	66 (7.1%)	5	991	4 (0.4%)	1	904	2 (0.2%)	1	[51]

The number of samples tested for each toxin, with the % presence of each toxin and the number of areas each toxin has affected are recorded. In 2006, LTs (Lipophilic toxins) were detected, which included OA/DTXs, AZAs, YTXs and PTXs.

**Table 5:** CEFAS water sample results for four toxic marine microalgae species from 1999-2009 in Wales and England

		*Dinophysis spp*.		*Prorocentrum spp*.		*Alexandrium spp*	*Pseudo-nitzschia*		
	
	No. water samples tested	% Present	%> limit	% Present	%> limit	% Present	% Present	%> limit	Reference
**April 1999 - March 2000**	320	NA	1 (0.3%)	NA	NA	4 (1.3%)	NA	0	[49]

**April 2000 - March 2001**	350	25 (7.1%)	2 (0.6%)	NA	NA	NA	15 (4.3%)	0	[56]

**April 2001 - March 2002**	195	NA	0	NA	NA	NA	14 (7.2%)	0	[57]

**April 2002 - March 2003**	1529	23 (1.5%)	1 (0.1%)	NA	NA	NA	50 (3.3%)	0	[58]

**April 2003 - March 2004**	388	0	0	NA	NA	13 ( 3.4%)	18 (4.6%)	0	[59]

**April 2004 - March 2005**	314	6 (1.9%)	1 (0.3%)	6 (1.9%)	1 (1.9%)	6 (1.9%)	28 (8.9%)	0	[50]

**April 2005 - March 2006**	737	23 (3.1%)	7 (0.9%)	3 (0.4%)	1 (0.1%)	80 (10.9%)	277 (37.6%)	3 (0.4%)	[60]

**April 2006 - March 2007**	879	83 (9.4%)	31 (3.5%)	10 (1.1%)	5 (0.6%)	150 (17.1%)	374 (42.5%)	32 (3.6%)	[61]

**April 2007 - March 2008**	1122	18 (1.6%)	4 (0.4%)	3 (0.3%)	0	139 (12.4%)	570 (50.8%)	4 (0.4%)	[62]

**April 2008 - March 2009**	1079	25 (2.3%)	4 (0.4%)	11 (1.0%)	5 (0.5%)	75 (6.9%)	438 (40.6%)	21 (1.9%)	[51]

The number of water samples tested, with the species % present in each sample, and the % of samples exceeding the action limit have been recorded. (NA = no results during that time period).

### UK Incidents

Although the potential adverse effects of toxic marine microalgae are well documented, there are very few epidemiological studies designed to thoroughly assess these effects [3]. Within the UK, there are few published records of shellfish poisonings, and we suspect that there is a high level of underreporting, as there is no set database which systematically records the number or frequency of incidents.

### Epidemiology of shellfish poisoning in the UK, 1960-2009

#### PSP

The first reliably reported case of PSP in the UK was on the East Coast in 1969, where high concentrations of *Alexandrium tamarense *were monitored up to 15 miles offshore [2,25]. This outbreak caused illness in 78 humans and was responsible for the death of numerous birds and other marine mammals in the region [2,26,27] (Table 6). In the UK, it is thought that PSP is regional in nature, occurring in particular hotspots mainly in Scotland, e.g. Orkney and Shetland Islands [4]. There appear to be, however, few reported cases of PSP affecting fisheries throughout the UK, with the longest closure occurring in Scotland during 2000-2001, with aquaculture and scallop fisheries affected by PSP throughout the year (Table 6).

**Table 6:** The history and consequences toxic events of dinoflagellate PSP toxin seafood poisoning within the UK from 1969-2007

Year	Event	Reference
1969	Toxins monitored up to 15 miles offshore on the East Coast of UK. Caused illness to 78 humans and was responsible for the death of numerous birds and marine mammals. Species responsible: *Alexandrium tamarense*.	[2,25-27]

1990	On the NE English coast in May high levels of toxins detected in mussels and scallops. Commercial fisheries were closed.	[25]

2000	TPO was placed in Falmouth in July, as PSP was above action limit. Species responsible was *Alexandrium*.	[56]

2000-2001	In Scotland, toxins detected throughout the year in aquaculture sites along the west coast, and in scallop fisheries grounds in Orkney and East coast Scotland. Led to restrictions in Fishing.	[66]

2001	Toxins found in scallops in the sea adjacent to Northern Ireland. Led to a ban on scallop fishing.	[66]

2002	Warning notices and VCA were places in Salcombe estuary, Devon, from July to November, and Holy Island, Northumberland, in May, when cockles and mussels returned positive results.	[58]

2002	Loch Eishort, in Skye and Lock Hourn, were closed in June and July where toxins were detected in mainly mussels.	[67]

2002-2003	PSP was detected in scallops in Orkney, Morary Firth, and the North Minch from May to September. Fisheries closures were implemented.	[67]

2005	Mussels from Pont Pill, Fowey found PSP toxins above the regularity limit. The site was subjected to temporary harvesting restrictions.	[60]

2006-2007	Toxins were detected in Two areas of the Fal, Cornwall. Temporary harvest restrictions in June and July.	[61]

(TPO = Temporary Prohibition Order. VCA = Voluntary Closure Agreement).

Throughout 1999-2009, the toxin PSP has maintained a low level in routine testing (average 0.73%), affecting between 1-4 sampling locations, within Wales and England (Table 4). The PSP producing species *Alexandrium *has also maintained a low abundance throughout Wales and England during 2005-2009 (Table 5).

#### DSP

DSP was first reliably recorded in the UK in 1997, when 49 patients showed symptoms 30 minutes after consuming mussels in two London restaurants [28]. Since then the incidents and the presence of DSP appears to becoming more frequent and prolonged [29], which may be partly due to increased knowledge and surveillance programmes. Table 7 shows 19 incidents from 1999-2009, over a wide range of areas throughout the UK. Temporary closures and voluntary closures lasted between a few weeks up to seven months.

**Table 7:** The history and consequences toxic events of dinoflagellate DSP toxin seafood poisoning within the UK from 1997-2009

Year	Event	Reference
1997	49 patients showed symptoms 30 minutes after consuming mussels in two London restaurants.	[28]

1999	Voluntary closure and warning signs for the general public was undertaken in Holly Island, Northumberland for 6 weeks as Pacific Oyster retuned a positive DSP result.	[49]

2000	TPO was induced from early February to March on the Northern side of the Solent, as Oysters returned positive results.	[56]

2000	Toxins detected in mussels from Cornwall, cockles from southeast England and from south Wales, led to harvesting restrictions.	[68]

2000	DSP was detected in England and Wales, with large scale closures (TPO and VCA) with the Solent shellfisheries from June - November, and the Thames shellfisheries from July - September.	[56]

2000	In July, toxins detected in Fleet Lagoon, Dorset. Harvesting was closed until 4^th ^September. Species responsible: *Prorocentrum lima*.	[30]

2000	DSP affected a large number of areas in Scotland. East coast between July-September. Orkney between July-August. 10 sites within Shetlands between July-October. Outer Hebrides in May, and July-October, and 28 locations in Clyde between May -December. Long term closures of shellfish farming in Scotland lasted up to 24 weeks.	[69]

2000-2001	Camel Estuary, Devon was closed from August until February as mussels returned positive DSP results.	[56]

2000-2001	Toxins detected in mussels and scallops and re-appeared at several sites throughout the year. Restrictions on harvesting at affected sites.	[68]

2001	DSP was detected in The Thames from June-September, and Blyth Northumberland in July, leading to TPO and VCA within shellfisheries.	[57]

2001	The Wash shellfisheries were subjected to closures over winter as DSP was detected in Cockles.	[57]

2001-2002	Cockles from Burry Inlet, Wales produced positive DSP results from June 2001 to March 2002. Leading to a long term closures of shellfisheries.	[57]

2001	Toxins found in scallops in the sea adjacent to Northern Ireland. Led to a ban on scallop fishing.	[66]

2002-2003	Burry Inlet, Thames Estuary and The Wash was subjected to harvest closures every month except May and November for Burry Inlet, September for the Thames, and September, October and February for the Wash.	[58]

2002	DSP toxins were detected throughout Scotland, where VCAs were placed in several regions. Majority of cases closures lasted for periods of four to six weeks, but some closures lasted up to seven months.	[67]

2005	DSP were detected in three areas, East of Ajax, Plymouth, The Wash, and Clamerkin Creek, Newtown. All were subject to temporary harvesting restrictions.	[60]

2006-2007	Toxins were detected in three regions in Cornwall, which led to temporary harvest restriction.	[61]

2007-2008	Toxins detected in Southampton Water, Fal River: Cornwall, Salcombe, Devon, which led to closed to temporary harvesting restrictions.	[62]

2008	Shetland Islands were affected by a large outbreak, closing 13 areas from April-October 2008.	[70]

2008-2009	Seven regions in Argyll and Bute, Scotland suffered temporary closures. With the West Loch Tarbert being closed from April 2008-Feburary 2009.	[70]

During 1999-2004, there was a high percentage of DSP toxin detected in shellfish samples (average of 13.5%) (Table 4). However, the level of LTs detected, which include newly identified toxins, has dramatically decreased, only averaging 2.1% from 2004-2009, within Wales and England. *Dinophysis spp*. has been detected in UK waters, from 2004-2009 (Table 5) and it is thought that *D. acuminata *and *D. acuta *are the main species that dominate, especially in Scottish waters [4]. During 2006-2007, 3.5% of *Dinophysis spp*. samples returned a greater than the action limit throughout Wales and England. The species *Prorocentrum *is also associated with DSP, and has been detected since 2004, but due to its epiphytic and epibenthic nature, it may be under-represented in sampling programmes [4,30].

#### AZP

Azaspiracids have been identified in mussels within the UK [21,31], although there have been no reported incidents of poisoning. However, mussels cultivated in Killary Harbour, Ireland, were responsible for the intoxication of at least eight people in the Netherlands in November 1995 [31] (Table 8). Since 1996, several other human intoxications have been reported in Ireland around the Arranmore Island region on Donegal, Northwest Ireland [32], and in 1997 AZP persisted in this region for seven to eight months [21]. In 2000, a number of food poisoning incidents occurred in the UK after the consumption of processed mussels which originated from the SW coast of Ireland. These mussels were initially deems safe-for-human consumption following negative MBAs, but it was later identified that AZP was he causative toxin [33].

**Table 8:** The history and consequences toxic events of dinoflagellate AZP toxin seafood poisoning within the UK from 1995-2000

Year	Event	Reference
1995	Mussels cultivated in Killary Harbour, Ireland, were responsible for the intoxication of at least eight people in the Netherlands in November.	[31]

1996	Human intoxications have been reported in Ireland around the Arranmore Island region on Donegal, Northwest Ireland.	[32]

2000	In August, a number of incidents of food poisoning occurred in Sheffield, Warrington, Alyesbury and the Isle of Wight after the consumption of processed mussels originating from the SW coast of Ireland. These mussels were deemed safe-for-human consumption following negative mouse bioassays. Later identified that AZP was the causative toxin.	[33]

#### ASP

ASP was first detected in the UK in Scotland (Shetland) in 1997, when traces of Domonic Acid (DA) were detected [34]. Since then, there have been several ASP outbreaks throughout the UK, causing temporary fisheries closures. There have been two very large outbreaks; in July 1999, when a scallop fishing area of 8,000 square miles was closed in the north west of Scotland following the discovery of ASP toxin over the regulatory limit. In 2002, offshore scallop grounds continually detected ASP throughout the year, leading to fisheries closures (Table 9).

**Table 9:** The history and consequences toxic events of diatom ASP toxin seafood poisoning within the UK from 1999-2003

Year	Event	Reference
1999	In July, a scallop fishing area of 8,000 square miles was closed in the north west of Scotland following the discovery of ASP toxins over the regulatory limit. Species responsible *Pseudo-nitzschia australis*.	[71,72]

1999	A TPO was taken in Poole Harbour on the 6 March. One mussel sample returned over 20 μm of DA.	[49]

2000	VCA of four aquaculture sites in Scotland as Scallops returned positive results for ASP.	[69]

2000-2001	Toxins detected in Scallops above the regulatory limit. Restrictions on fishing activities were placed on affected regions in Scotland.	[68]

2002	ASP was detected at Dale Voe, Shetlands in September, in Loch Moidart during in July, and Broadford Bay in July. Harvesting restrictions were imposed as necessary.	[67]

2002-2003	Offshore scallop grounds in Scotland, continually detected ASP throughout 2002 and early 2003. Fisheries closures were implemented in affected shellfisheries.	[67]

During 1999-2000 and 2000-2001, ASP returned a positive result in 6.1% of samples affecting 21 areas, and 8.8% effecting 32 areas respectively (Table 2). Since then, the percentage of positive results has remained between 0.2 - 2.6%. However, *Pseudo-nitzschia *has showed a dramatic increase in abundance since 2005, with an average of 42.9% of water samples returning a positive result (Table 4). During 2006-2007, and 2008-2009, 3.6%, and 1.9% samples respectively were greater than the regulatory limit (> 150,000 cells/Litre) (Table 5).

Despite the increase in *Pseudo-nitzschia *in recent years, which has been shown in both the CEFAS water samples and the CPR, the level of toxin is not representative. Suggesting that toxin production might not be dependent on the abundance of the species. Water samples showed ASP was at its highest when the percentage of *Pseudo-nitzschia *was at its lowest (Table 5), implying that stressful conditions could cause a greater increase in toxin presence.

### Routine clinical records (NHS) survey, 1998-2009

The reporting of shellfish poisoning incidents to date has been sporadic. To attempt a systematic review, we accessed hospital records in Wales using the Secure Anonymised Information Linkage (SAIL) databank [35]. The important step here is the anonymous linkage of hospital events to a very wide set of health information to the individual, including demographics, mortality statistics, GP records and laboratory tests. This potentially allows a very detailed assessment of the hospital event, and confirmation of its cause and longer term effects. In brief, a split-file approach is used to ensure anonymisation. The datasets are prepared by Health Solutions Wales and separated into, clinical and demographic data. An anonymous system linking field (ALF) is assigned to ensure that the data can be re-connected later at the analysis stage [36,37]. Clinical data includes information on diagnostic tests, therapeutic tests and interventions. Demographic data is comprised of person based variables, such as gender and age.

First, all hospital episodes that had any mention of "Toxic effects of noxious substances eaten as seafood (ICD10 code T61)" for all Welsh NHS Trusts during April 1998 - 31^st ^August 2009 were identified. From 1998-2009, there were 61 hospital episodes within Wales, with 5 of those being re-admission following an initial visit. Out of the 56 individual hospital patients, 51 were successfully allocated an ALF, which enables the anonymous linkage of person level data within and across all national datasets. From the 51 ALF admissions, 7 detailed pathology reports, and 6 mortality records from 2003-2009, were identified.

GP clinical information recorded within +/- 30 days of the indexed hospital episode was identified for 10 of the patients.

### Interpretation of routine clinical records

Within Wales, 56 individual patients were identified with "Toxic effects of noxious substances eaten as seafood" from 1998-2009, with an age range of 5-94 years. The length of stay in hospital varied between 1 to 11 days, with the average stay length of 2.5 days (significantly related to age). The majority of incidents occurred during the summer months (June-August). Six patients were noted to have died, with a delay of between 7 months and 9 years after their incident, suggesting that there have been no deaths in Wales directly resulting from shellfish poisoning over the survey period. The population of Wales is approximately 2.9 million, hence assuming these patterns are representative, these results imply an estimated incidence of shellfish poisoning of 100 cases per year in the UK (of 16 per million per year).

These estimates however must be treated with caution. The clinical presentation may be non-specific, since viral infections, particularly norovirus, or allergy, can cause similar gastrointestinal symptoms to those of shellfish toxin poisonings. A definitive diagnosis is only possible where samples of suspect food are available and tested for toxin, which happens rarely as the food has usually been consumed or discarded before a formal investigation can begin, unless part of a wider epidemiological investigation. Timing of symptom onset is the most helpful factor in distinguishing toxin-induced (very rapid onset) from viral (several hours) causation. Our dataset does not contain this information, although negative results were noted for all bacteriology/virology tests, where they had been performed. Two patients were also tested for the antibody Immunoglobulin E (IgE) to chub mackerel, where low traces were detected, potentially indicating that the patients suffered from an allergic reaction to chub mackerel. It has been shown that chub mackerel are lethal vectors for PSP toxins (saxitoxin (STX), and gonyautoxins) all year around [38], suggesting a possible toxin involvement in these two cases.

Therefore in many cases, it is difficult to be sure whether a case coded as 'toxin poisoning' was not in fact viral gastroenteritis, or allergy - where a medical diagnosis of "food poisoning following shellfish" has been made this may or may not be inputted as toxin-related by the final coder. Furthermore, mild cases are likely to go unreported.

We conclude that although the incidence of shellfish poisoning is likely to be low, the current data is insufficient to allow an accurate estimate. This raises the important question of how changes in the incidence rate, that might be associated with the distribution of toxic microalgae of HAB species, would be detected? At the human level, although it is a notifiable event, reporting is not likely to be accurate enough to quickly identify trends over time. In contrast, routine testing at the fisheries level is much more likely to be able to identify changes in the rate of toxic events and remains the key surveillance system in the UK, acting as a sentinel for potential human impact.

### The future of key indicator toxic microalgae species in UK waters

Recent studies suggest that some toxic microalgae species are increasing in frequency and geographical location on a global scale [3]. It is thought that human assistance has spread some species through a variety of mechanisms e.g. ballast water transfer, increase in eutrophication, and aquaculture development [26,39]. However, the establishment and reoccurrence of blooms cannot occur without a hospitable environment [40]. There have been several suggestions that an alternative or additional explanations for the spreading of some species are as a result of the effects of changing currents, weather patterns, and changing ocean temperatures associated with global warming [8,41], allowing species to occupy regions in which they would not normally survive.

There is increasing evidence in the literature of the effects of climate change on the phenology of marine organisms. Estimates show that British winters now end 11 days earlier on average than in the mid 1970s [42]. These changes in phenology have caused dramatic shifts in the timing and occurrence of species during the year with a pole-ward shift in latitude distribution range [43], in response to the changing environmental conditions. The UK could gain species from an equatorial direction, and lose existing species as conditions become too warm [43]. In the North Sea, total species abundance has remained relatively stable but the species composition has changed [44]. Warm-water species have increased, while colder-water species have decreased, e.g. euphausiids [45] owing to the warming sea temperatures and changes in climate indices such as the North Atlantic Oscillation Index [46]. From the CEFAS water samples results, we have already noticed a change in species abundance with *Pseudo-nitzschia *now being present in 37-51% of samples during 2005-2009 (Table 5). This increase in *Pseudo-nitzschia *has also been observed (personal observation), using the Continuous Plankton Recorded. There has been an expansion of the geographic range and a lengthened seasonal window of *Pseudo-nitzschia seriata *in the North Atlantic and North Sea during 2000-2009.

Although there has been few human related illness from ingestion of toxins from shellfish in the UK, there remains a threat that this risk will increase over time. The potential future change in species composition due to climate change, could bring new toxic species into the surrounding UK waters. Unless these potential toxic marine species are monitored in their distribution and frequency, these new species could go unnoticed by the current monitoring system. If that is the case there could be several more human related incidents within the UK, whether it is medical related or indirect, via the closure of aquaculture sites. In contrast, in the USA, estimates of the economic impact of HABs are averaged at $75 million/year over the period 1987-2000, which includes impacts from public health, commercial fishing, recreation and tourism, and monitoring and management costs [47]. PSP is the most severe of the toxin syndromes, with a total of 500 cases and 30 deaths reported in California, since 1927 (a mortality rate ranging between 1-12%, [48]), caused in part by poor access to advanced life support capabilities [48].

While the geographic distributions of species are clearly important, we also note that the abundance of a species does not necessarily correlate strongly to the amount of toxin produced. There have been several CEFAS reports, where there was high toxin content detected, but water samples showed a low species abundance [49,50]. This was especially noticeable for the toxin causing ASP, where the highest levels of toxin detected was when *Pseudo-nitzschia *was at its lowest abundance. The mechanisms behind toxin production are not fully understood, and it is thought that stress could be an initial trigger. If that is the case, toxin production within the UK could potentially increase as the current UK HAB species have to adapt to the changing hydrological conditions.

## Conclusions

We have shown that within UK waters, outbreaks of shellfish poisoning are occurring on a regular basis in different regions and affect a diverse range of molluscan shellfish and other marine organisms. Every year, several aquaculture site and shellfisheries are closed due to shellfish toxins, with closures ranging from weeks to several months. The toxin syndrome DSP has shown to be the most problematic toxin within the UK, with 19 records during the period 1999-2009, which resulted in several shellfish harvesting closure. However, despite the regular occurrence of shellfish poisonings, data on human epidemiology of poisoning linked to toxic marine microalgae species is sparse. An extensive literature review of shellfish poisoning has shown that only disparate records are available for incidents and episodes within the UK, with only the large outbreaks related to shellfish and aquaculture industries being recorded. However, the accuracy of the UK data must be treated with caution. Despite obtaining data for hospital admissions, GP records, and pathology records, it remains extremely difficult to determine the precise number of patients who have been affected by toxic shellfish poisoning.

In the absence of a suitable epidemiological surveillance system, the routine fisheries testing regimes remain the key indicator of any potential change in exposure of the human population. From this routine testing we have already seen an increase in abundance of *Pseudo-nitzschia *in the last 5 years with the species now being present in over 50% of the water samples. In addition, predicted warmer waters in the UK could suit several species with southern biogeographical affinities enabling them to occupy the coastal regions of the UK, but which are not yet considered to be detrimental. To ensure the UK monitoring systems are up to date with the increasing number of new toxic species and the changing seasonal and geographic distribution patterns, research into the environmental conditions that lead to bloom development and toxin production would help predict bloom event. Long term time series of toxic marine microalgae need to be monitored to assess and determine if species are indeed spreading in their geographical location or increasing in frequency.

## List of Abbreviations

ALF: Anonymous System Linking Field; ASP: Amnesic Shellfish Poisoning; AZP: Azaspiracid Poisoning; CEFAS: Centre for Environment, Fisheries and Aquaculture Science; CFP: Ciguatera Fish Poisoning; DA: Domonic Acid; DSP: Diarrhetic Shellfish Poisoning: DTX: Dinophysis Toxins; FSA: Food Standards Agency; HAB: Harmful Algal Blooms; HIRU: Health Information Research Unit for Wales; HPLC: High Performance Liquid Chromatography; LC-MS: Liquid Chromatography - Mass Spectrometry; LT: Lipophilic Toxin; MBAs: Mouse Bioassays; NSP: Neurotoxic Shellfish Poisoning; OA: Okadaic Acid; PSP: Paralytic Shellfish Poisoning; PTXs: Pectenotoxin; SAIL: Secure Anonymised Information Linkage; STX: Saxitoxin; TPO: Temporary Prohibition Order; VCA; Voluntary Closure Agreement; YTXs: Yessotoxin.

## Competing interests

The authors declare that they have no competing interests.

## Authors' contributions

GCH and MG designed the study. SLH summarised the historical data available. MG, CB and SLH obtained the HIRU (SAIL) data for Wales. APD, MG and SLH examined the HIRU dataset. SLH and MG drafted the manuscript. All authors read and approved the final manuscript.
